# Case Report: Multimodal Functional and Structural Evaluation Combining Pre-operative nTMS Mapping and Neuroimaging With Intraoperative CT-Scan and Brain Shift Correction for Brain Tumor Surgical Resection

**DOI:** 10.3389/fnhum.2021.646268

**Published:** 2021-02-25

**Authors:** Suhan Senova, Jean-Pascal Lefaucheur, Pierre Brugières, Samar S. Ayache, Sanaa Tazi, Blanche Bapst, Kou Abhay, Olivier Langeron, Kohtaroh Edakawa, Stéphane Palfi, Benjamin Bardel

**Affiliations:** ^1^Department of Neurosurgery, DMU CARe, Henri Mondor University Hospital, Assistance Publique – Hôpitaux de Paris (APHP), Creteil, France; ^2^Translational Psychiatry (Equipe 15), IMRB – INSERM U955, Univ Paris-Est Creteil, Creteil, France; ^3^Department of Clinical Neurophysiology, DMU FIxIT, Henri Mondor University Hospital, Assistance Publique – Hôpitaux de Paris (APHP), Creteil, France; ^4^Excitabilite Nerveuse et Therapeutique, EA 4391, Univ Paris-Est Creteil, Creteil, France; ^5^Department of Neuroradiology, DMU FIxIT, Henri Mondor University Hospital, Assistance Publique – Hôpitaux de Paris (APHP), Creteil, France; ^6^Department of Anesthesiology and Critical Care, DMU CARe, Henri Mondor University Hospital, Assistance Publique – Hôpitaux de Paris (APHP), Creteil, France; ^7^Experimental Neuropathology Unit, Institut Pasteur, Paris, France; ^8^Department of Neurosurgery, Graduate School of Medicine Osaka University, Suita, Japan

**Keywords:** brain tumor surgery, case report, resting state fMRI, navigated TMS, tractography

## Abstract

**Background:** Maximum safe resection of infiltrative brain tumors in eloquent area is the primary objective in surgical neuro-oncology. This goal can be achieved with direct electrical stimulation (DES) to perform a functional mapping of the brain in patients awake intraoperatively. When awake surgery is not possible, we propose a pipeline procedure that combines advanced techniques aiming at performing a dissection that respects the anatomo-functional connectivity of the peritumoral region. This procedure can benefit from intraoperative monitoring with computerized tomography scan (iCT-scan) and brain shift correction. Associated with this intraoperative monitoring, the additional value of preoperative investigation combining brain mapping by navigated transcranial magnetic stimulation (nTMS) with various neuroimaging modalities (tractography and resting state functional MRI) has not yet been reported.

**Case Report:** A 42-year-old left-handed man had increased intracranial pressure (IICP), left hand muscle deficit, and dysarthria, related to an infiltrative tumor of the right frontal lobe with large mass effect and circumscribed contrast enhancement in motor and premotor cortical areas. Spectroscopy profile and intratumoral calcifications on CT-scan suggested an WHO grade III glioma, later confirmed by histology. The aforementioned surgical procedure was considered, since standard awake surgery was not appropriate for this patient. In preoperative time, nTMS mapping of motor function (deltoid, first interosseous, and tibialis anterior muscles) was performed, combined with magnetic resonance imaging (MRI)-based tractography reconstruction of 6 neural tracts (arcuate, corticospinal, inferior fronto-occipital, uncinate and superior and inferior longitudinal fasciculi) and resting-state functional MRI connectivity (rs-fMRI) of sensorimotor and language networks. In intraoperative time, DES mapping was performed with motor evoked response recording and tumor resection was optimized using non-rigid image transformation of the preoperative data (nTMS, tractography, and rs-fMRI) to iCT data. Image guidance was updated with correction for brain shift and tissue deformation using biomechanical modeling taking into account brain elastic properties. This correction was done at crucial surgical steps, i.e., when tumor bulged through the craniotomy after dura mater opening and when approaching the presumed eloquent brain regions. This procedure allowed a total resection of the tumor region with contrast enhancement as well as a complete regression of IICP and dysarthria. Hand paresis remained stable with no additional deficit. Postoperative nTMS mapping confirmed the good functional outcome.

**Conclusion:** This case report and technical note highlights the value of preoperative functional evaluation by nTMS updated intraoperatively with correction of brain deformation by iCT. This multimodal approach may become the optimized technique of reference for patients with brain tumors in eloquent areas that are unsuitable for awake brain surgery.

## Introduction

Maximum safe resection of infiltrative brain tumors in the eloquent areas is the primary objective in surgical neuro-oncology. Intraoperative direct electrical stimulation (DES) is the standard of care to map motor cortex function (Rossi et al., 2019; Duffau, 2020), and preserve speech and cognitive functions during surgery, when it is feasible in awake patients (Gogos et al., 2020). However, there are still contraindications for awake craniotomy (AC), such as increased intracranial pressure (IICP), uncontrolled seizures, or important motor, speech cognitive or psychiatric deficits impeding the patients' ability to perform intraoperative tasks. For patients with a brain tumor in an eloquent area and who are not eligible for AC, there is a critical need for innovative operative mapping techniques (Ille et al., 2016; Raffa et al., 2018) before and during general anesthesia (GA). Through this case report, we propose a pipeline procedure combining advanced techniques aimed at performing a dissection that respects the anatomo-functional connectivity of the peritumoral region under GA. This procedure is composed of a multimodal preoperative mapping including navigated transcranial magnetic stimulation (nTMS) and various neuroimaging modalities (tractography and resting state functional MRI, rs-fMRI). The distinctive feature of this pipeline is the use of intraoperative mapping with navigated DES (nDES), but also intraoperative computerized tomography scan (iCT-scan) with brain shift correction (BSC). This technical novelty allows the neurosurgeon to use data from non-invasive anatomo-functional preoperative mapping (M) and merge them with iCT-scan data and BSC throughout the entire course of the surgery in the operating room (OR). Therefore, this procedure was called M-iCT-BSC.

## M-iCT BSC Surgery Pipeline and Workflow Diagram

The pipeline can be divided into several key steps:

1. Pre-operative functional cortical mapping

The pre-operative mapping was performed using nTMS to achieve a precise, reproducible cortical motor mapping of several affected and unaffected muscles of the limbs contralateral to the location of the brain tumor.

2. Pre-operative structural and functional imaging

Beside usual MRI brain sequences, a diffusion tensor imaging was used to present a thorough reconstruction of all white matter tracts of interest. A tractography reconstruction of the pyramidal tract was guided by nTMS (nTMS-based/seed-based functional tractography). The functional location of the primary motor cortex (M1) as defined by nTMS was used as region of interest (ROI) for tractography reconstruction, in place of the standard anatomical location of M1, which is severely distorted as a result of tumor infiltration or perilesional edema. On the other hand, the five main speech tracts were reconstructed using ROI-based tractography by an experienced neuroradiologist.

In addition, functional brain connectivity of rs-networks was appraised by rs-fMRI. This approach is especially helpful to identify functional brain networks in patients with neurological impairments who would be unable to comply with complex tasks.

3. Intraoperative iCT-scan with BSC

This correction was performed at crucial steps, i.e., after opening the dura and halfway during tumor resection.

- The first step was to implement all the aforementioned data (nTMS, nTMS-based tractography and rs-fMRI) in the iCT before craniotomy in the OR.
- A second step was to acquire iCT during surgical resection when a significant brain shift was likely to have occurred.
- Then, elastic fusion of the iCT to the preoperative brain MRI was performed for BSC.
- Preoperative nTMS as well as motor nTMS-guided tractography, speech anatomical tractography and motor and speech rs-fMRI data were updated in the virtual MRI with BSC. This procedure allowed the neurosurgeon to keep performing a high-resolution morphological image-guided surgery through virtual MRI.
- Finally, nDES was performed cortically and subcortically to optimize the extent of the resection with minimization of motor function deficit.

**Figure 1:**
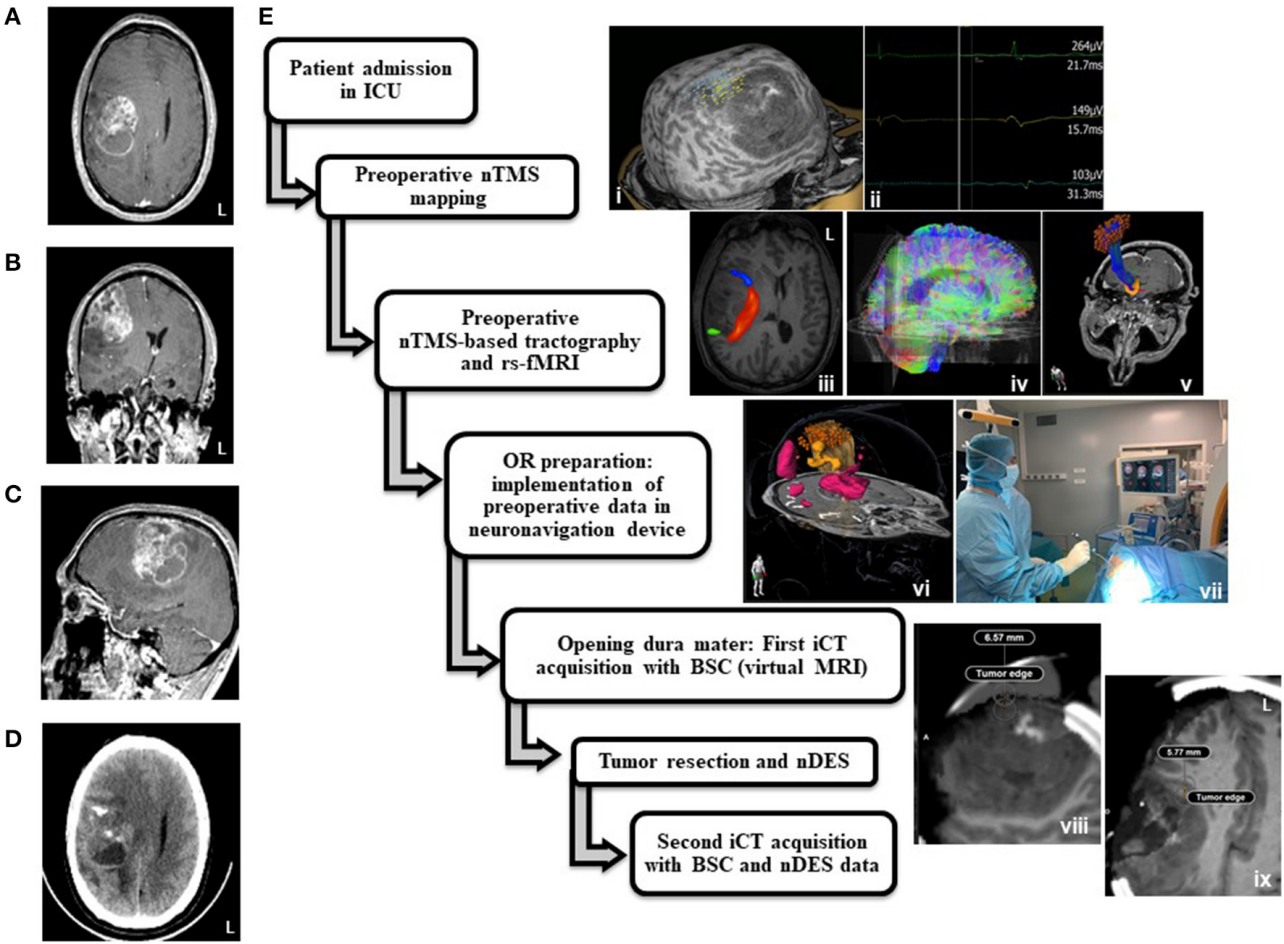
Medicosurgical management of the left-handed patient. Preoperative brain Magnetic Resonance Imaging (MRI): axial **(A)**, coronal **(B)**, and sagittal **(C)** slices of T1 weighted imaging after gadolinium intravenous injection, showing a frontal precentral right mass with heterogeneous contrast uptake and necrosis. Axial slice of Preoperative brain computerized tomography **(D)** showing intratumoral calcifications. Application of the “M-iCT BSC” procedure in this presented case **(E)**. i: navigated transcranial magnetic stimulation (nTMS) preoperative mapping (left panel) and Motor Evoked Responses examples (right panel) at 55% MSO for the TA (blue dots) and deltoid (yellow dots) muscles, and 81% for FID (green dots), ii: motor nTMS evoked potentials, iii: resting state functional MRI (rs-fMRI) region of interest (ROI) for right anterior speech network (blue), rs-fMRI ROI for right posterior speech network (green), arcuate/superior longitudinal fasciculus anatomical tractography reconstruction, iv: whole brain tractograms, v: motor nTMS-guided functional tractography, vi: 3D implementation of nTMS-guided motor tractography (yellow streamlines), anatomical speech tracts (white streamlines), motor rs-fMRI (orange ROI), speech rs-fMRI (pink ROI) and motor positive points for nTMS (orange dots). vii: Neuronavigation device, intraoperative monitoring device and intraoperative CT scan. viii: Axial slice of iCT the first intraoperative brain CT (iCT) acquisition with virtual MRI and brain shift correction (BSC). ix: Axial slice of second iCT acquisition with virtual MRI and BSC halfway through surgery.

The application of this pipeline in the timeline of care for the reported case is displayed in Figure 1.

## Case Description

A 42-year-old left-handed man presented to the emergency unit of our hospital (Henri Mondor Hospital, Créteil/France) with left hand paresis, left upper limb partial motor seizures and headaches that started 1 month earlier. The patient had a history of epilepsy diagnosed when he was 32, followed by his general practitioner without further neuroimaging exploration or antiepileptic drug prescription. Clinically, the neurosurgeon on call found left hand muscle deficit (3/5), dysarthria, and an IICP syndrome with clouding of consciousness, headache, nausea and vomiting.

An urgent brain MRI showed a 6 cm long infiltrative tumor of the right frontal lobe with tissular and kystico-necrotic portions, associated with large mass effect and circumscribed contrast enhancement in motor and premotor cortical areas.

The spectroscopy profile and intratumoral calcifications on CT-scan suggested an WHO grade III glioma (Figures 1A–D). During the weekly neurooncological tumor board, it was agreed with the anesthesiologists in care that the IICP syndrome including clouding of consciousness contraindicated AC and therefore the tumor would be removed under GA. The main objective was the preservation or even the postoperative improvement of the function of the left hand of the patient who was a computer engineer. He gave his informed consent for the report of his case to be published in medical literature.

### Navigated Transcranial Magnetic Stimulation (nTMS) Mapping (Figure 2a)

Prior to surgery, nTMS mapping of motor function was performed using Navigated Brain Stimulation (NBS) system (Nexstim, Helsinki, Finland). The 3D T1-weighted brain MRI was co-registered to the patient's head using anatomical landmarks and surface matching. EMG surface electrodes (Natus, Paris, France) were placed on deltoid, first dorsal interosseous (FDI), and tibialis anterior (TA) muscles of the right side with an additional large adhesive ground electrode placed over the forearm. Cortical mapping was performed according to nTMS Workshop Report (Krieg et al., 2017), with additional stimulation points at the edge of the tumor, 20 mm deep from the scalp surface (peeling depth). An initial step of rough mapping in the anatomical cortical motor area was done to determine the hand motor hotspot, i.e., the location eliciting the largest motor evoked potentials (MEPs) for the FDI. Since the resting motor threshold (RMT) was higher than usual for the FDI (74%), we decided to perform a motor mapping at only 110% of RMT, the RMT being determined individually for each muscle. As a result, the maximal stimulator output (MSO) used for motor mapping was 81% for FDI, and 55% for both TA and deltoid. The nTMS points eliciting MEP responses were exported at a combined peeling-depth of 15, 20, and 25 mm in the 3D-T1 weighted space.

**Figure 2:**
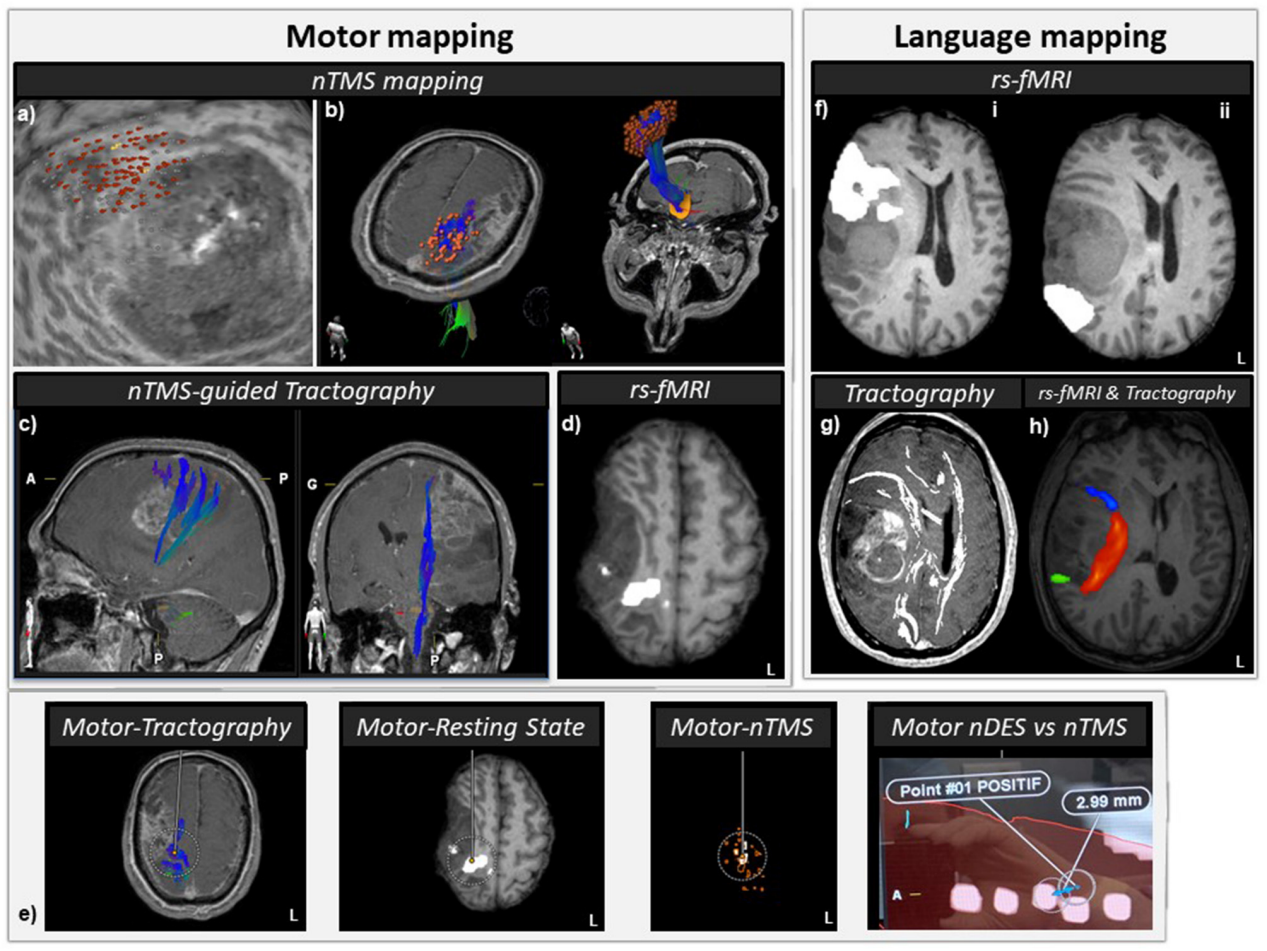
Anatomo-functional peritumoral preoperative mapping. **(a)** nTMS preoperative motor mapping: red dots represent positive nTMS sites, i.e., where a single pulse TMS elicited a motor evoked potential (MEP) (peak-to-peak amplitude above 50 μV). The medial and posterior cortical edges of the tumor are at risk during surgery regarding motor function. **(b)** nTMS-guided functional tractography reconstructions: orange dots stand for positive nTMS sites nTMS, used as seeds to perform functional tractography (red-green-blue tracts). **(c)** Subcortical display of the motor nTMS-guided tracts located in the vicinity of the medial and posterior edges of the tumor. **(d)** Resting state functional MRI (rs-fMRI) regions of interest (ROIs) for motor function (white) superimposed on a T1WI axial slice. The posteromedial edge of the lesion seems at risk for motor function during surgery. **(e)** Peritumoral multimodal motor mapping: preoperative nTMS mapping, nTMS-guided tractography, rs-fMRI, and intraoperative neuronavigated direct electrical stimulation (DES) of the cortical and subcortical regions. Distance between intraoperative neuronavigated positive DES locations and preoperative multimodal positive locations can be accurately measured and then, compensated for brain shift (cf Figure 3). After brain shift correction (BSC), distance between DES at 3 mA and closest nTMS motor positive point was 2.99 mm. **(f)** Rs-fMRI ROIs (in white) for anterior speech (i) and posterior speech function (ii) superimposed on a T1WI axial slice. The anterior and posterior edges of the lesion might be at risk for speech function during surgery. **(g)** Anatomical tractography reconstructions of five speech tracts (arcuate (AF)/superior longitudinal (SLF), inferior fronto-occipital (IFOF), frontal aslant (FAT), uncinate (UF), and inferior longitudinal (ILF) fasciculi. **(h)** Rs-fMRI ROIs for right anterior speech network (blue), rs-fMRI ROIs for right posterior speech network (green), and arcuate/superior longitudinal fasciculus (AF/SLF) anatomical tractography reconstruction. The AF/SLF tract connects some subregions of the anterior and posterior speech rs-fMRI ROIs.

In addition to an elevated RMT of the FDI compared to deltoid and TA, the nTMS cortical mapping revealed a smaller FDI cortical area compared to the deltoid and TA suggesting an hypo-excitability and/or a loss of corticospinal neurons. Most of the FDI-MEP responses were elicited at the posterior edge of the tumor. Deltoid cortical map (CM) was represented at the normal-appearing posteromedial edge of the tumor in the posterior part of the precentral cortex. TA-CM was also equally represented in the T1 hypointensity parasagittal cortex (superior to the FDI-CM) and in the normal-appearing posterior part of the superior frontal gyrus.

### MRI Procedure, Tractography, and rs-fMRI

MR imaging was performed on a 3T Verio (Siemens, Erlangen, Germany) imager with a 32-channel head coil. Structural image was acquired using a 3D magnetization-prepared, rapid acquisition gradient echo (MP-RAGE) sequence with the following parameters: TR: 2,400 ms, TE: 3.65 ms, TI: 1,000 ms, flip angle: 8°, voxel size: 1 × 1 × 1 mm^3^, 190 slices.

Diffusion tensor imaging was acquired with the following parameters: TR: 8,033 ms, TE: 91 ms, 60 transverse sections of 2.5 mm thickness acquired parallel to the anterior commissure/posterior commissure line, number of averages: 1, imaging matrix: 122 × 122 (nominal resolution, 1.967 mm), and acceleration factor: 2. Diffusion weighting was encoded along 12 independent orientations using a b-value of 1,000 mm^2^/s. For speech tracts (Figures 2g,h), we used the methods described by Wakana et al. (2007) using both seed and target ROIs. For tracking the arcuate fasciculus on the directionally encoded tensor map, a coronal slice was selected at the middle of the posterior limb of the internal capsule. The seed ROI was placed in the deep white matter around the superior longitudinal fasciculus visible as a green triangular shape in the coronal plane. The target ROI was drawn on the axial slice at the anterior commissure level, around the descending portion of the superior longitudinal fasciculus seen as a blue structure. The uncinate fasciculus was tracked by selecting the most posterior coronal slice in which the temporal lobe was separated from the frontal lobe. The first ROI included the entire temporal lobe and the second ROI included the entire projections toward the frontal lobe. For tracking the inferior fronto-occipital fasciculus, the seed ROI delineated the occipital lobe on the coronal slice located between the posterior edge of the cingulum and the posterior edge of the parieto-occipital sulcus. For the target ROI, the entire hemisphere was delineated on a coronal slice at the anterior edge of the genu of corpus callosum. For tracking the inferior longitudinal fasciculus on a parasagittal slice, the posterior edge of the cingulum was located, and a coronal slice was selected at that edge. The first ROI included the entire hemisphere. The second ROI included the entire temporal lobe at the level of the most posterior coronal slice on which the temporal lobe was not connected to the frontal lobe. To dissect the frontal aslant tract, the seed region of interest was located in the white matter of the inferior frontal gyrus and the second region of interest in the white matter of the superior frontal gyrus. To determine the right corticospinal tract, we performed nTMS-guided tractography reconstructions (Krieg et al., 2012) using the nTMS motor ROI as seeds. The tractography was reconstructed with Brainlab fiber-tracking element software (BrainLAB AG, Munich, Germany) using a track angle threshold of 30° and a fiber assignment by continuous tracking propagation algorithm (Mori and van Zijl, 2002) (Figures 2b,c).

Whole brain rs-fMRI data were acquired using a T2^*^-weighted GE-EPI sequence with the following parameters: TR: 2,500 ms, TE: 24 ms, 180 volumes, voxel size: 2.5 × 2.5 × 2.5 mm^3^, 39 interleaved slices, flip angle: 80°, BW: 1,900 Hz/pixel, acceleration factor: 2, acquisition duration: 8 min. During the resting state acquisition, the patient was instructed to keep closed eyes and not to think of anything in particular. For rs-fMRI pre-processing, Matlab R2020b (MathWorks, Natick, MA, USA) and statistical parametric mapping SPM12 software (Wellcome department of imaging neuroscience, London, UK) were used for image pre-processing.

The processing included slice-timing correction, head motion correction, gray/white matter/cerebrospinal fluid structural segmentation. EPI images were coregistered to anatomical images and a spatial smoothing using a Gaussian filter with a full width at half maximum of 8 mm was then performed. Seed-based correlation analyses were performed using the CONN toolbox, which is an open-source Matlab/SPM-based cross-platform software for the analysis of functional connectivity (Whitfield-Gabrieli and Nieto-Castanon, 2012). This toolbox performs seed-based analysis by computing the temporal correlation (bivariate correlation) between the mean blood-oxygen-level-dependent (BOLD) signals from a given ROI to all other voxels in the brain. To remove possible sources of confounds present in BOLD signal data, all fMRI time-series underwent signal compensation from the ventricles, deep white matter and head motion, temporal filtering (0.009–0.08 Hz) on unsmoothed volumes. Seed to voxel connectivity maps were generated using motor and language ROIs as seeds. The motor and language subject-specific ROIs were chosen from the atlases of the CONN toolbox.

This multimodal preoperative mapping showed that motor function was especially at risk in the cortical, subcortical posterior, and subcortical medial areas, in the immediate vicinity of the tumor (Figures 2a–e). The language regions at risk were located on the anterior, posterior, and medial aspects of the tumor (Figures 2f–h).

### Surgical Procedure: nDES Mapping, BSC, and Resection

All pre-operative mapping data were implemented in the Brainlab navigation system in the OR. Craniotomy was performed after the patient was sedated by total intravenous anesthesia based on propofol and opioid infusion, as recommended for performing intraoperative MEP monitoring (Macdonald et al., 2013). In addition, short-duration neuromuscular blocking drugs were only used during endotracheal intubation.

**Figure 3:**
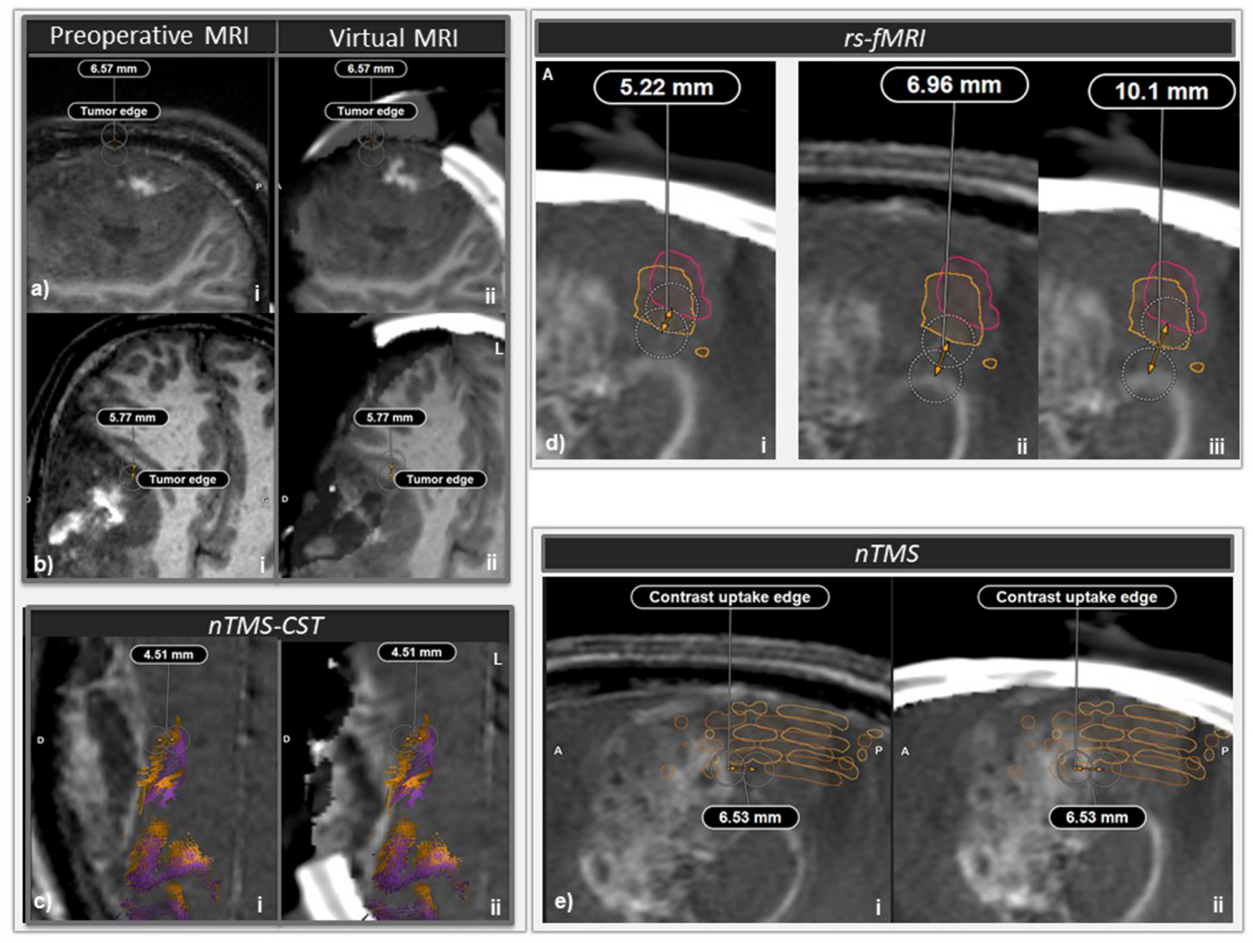
Intraoperative brain imaging and brain shift correction (BSC). **(a)** First intraoperative brain CT (iCT) acquisition and BSC, just after dura mater opening. i: sagittal slice of pre-operative magnetic resonance imaging (MRI), ii: sagittal slice of iCT and elastically transformed pre-operative MRI (virtual MRI) superimposed and coregistered with i. A significant tumor bulge occurred through the craniotomy and a brain shift of 6.57 mm could be measured at some location. **(b)** Second iCT acquisition with BSC halfway through surgery. i: Axial slice of preoperative MRI, ii: axial slice of iCT with virtual MRI. A 5.77 mm brain shift was measured at the level of the anteromedial edge of the tumor. **(c)** nTMS-guided corticospinal tract (nTMS-CST) comparison before (orange tracts) and after (purple tracts) BSC. i: Axial slice of preoperative MRI, ii: axial slice of second iCT acquisition with virtual MRI. At some location a 4.51 mm shift was measured between preoperative nTMS-MT before and after BSC. **(d)** Comparison of rs-fMRI ROIs before (orange outline) and after (pink outline) BSC. i and iii: sagittal slice of second iCT acquisition with virtual MRI, ii: sagittal slice of pre-operative MRI. At some location, a 5.22 mm shift was measured between preoperative rs-fMRI ROIs before and after BSC (i). At some location, the minimal distance between tumoral border and the preoperative rs-fMRI ROI shifted from 6.96 mm (ii) to 10.1 mm after BSC (iii). **(e)** Sagittal slice of pre-operative MRI (i) and second iCT acquisition with virtual MRI (ii). At some location, a 6.53 mm shift could be measured at some location chosen on the contrast uptake edge of the posterior aspect of the tumor between preoperative MRI and second iCT with virtual MRI. After elastic fusion and virtual MRI, nTMS positive ROIs shifted posteriorly relatively to the tumor.

Image guidance by neuronavigation included a correction for brain shift and tissue deformation at two crucial steps of the surgery. For this purpose, intraoperative imaging was acquired with the Airo® mobile CT scanner (BrainLAB) and the BSC was performed with elastic fusion using the Brainlab virtual MRI software (BrainLAB) with biomechanical simulation based on a finite element model taking into account the elastic properties of various brain regions (Riva et al., 2020). The first correction was applied when the tumor bulged through the craniotomy after dural opening: maximal bulge of the brain out of the craniotomy was measured as 6.57 mm (Figure 3a). The second correction was applied when approaching the presumed eloquent brain regions, reaching the medial, anterior, and posterior portions of the resection cavity, i.e., halfway through tumor resection: brain shifts of 5.77, 4.51, and 5.22 mm were found in the anterior portion of the remaining tumor (Figure 3b), for the nTMS-guided corticospinal tract (Figure 3c), and the motor rs-fMRI ROI (Figure 3d), respectively. Relative positions of tumor and motor nTMS ROI were also affected by brain shift (Figure 3e).

After the second BSC, we performed nDES mapping using a disposable bipolar concentric stimulation probe (Inomed, Emmendingen, Germany) and a Nimbus stimulator (Innopsys, Carbonne, France) combined with Brainlab neuronavigation tracking device (Brainlab, Germany) and keeping a bispectral index superior to 55. The DES settings were as usual (frequency: 60 Hz, pulse width: 1,000 μs, intensity: 3 mA) (Pallud et al., 2017). Motor responses were recorded using needle electrodes placed in the left deltoid, biceps, FDI, quadriceps, and TA muscles.

We continued the resection anteriorly guided by brain shift corrected anatomical MRI, speech anterior rs-fMRI data and speech tractography. Regarding the posterior and medial portions of the tumor, we were guided by neuroimaging data as well, but we ultimately respected the functional limits given by nDES and stopped tumor resection even though the parenchyma seemed visually infiltrated.

The updated neuronavigation data with BSC allowed us to directly compare discrepancy between motor cortical mapping obtained with preoperative nTMS and intraoperative nDES (Figure 2e). In this case, after BSC, a distance between DES at 3 mA and the closest nTMS motor positive point was 2.99 mm.

### Outcomes

**Figure 4:**
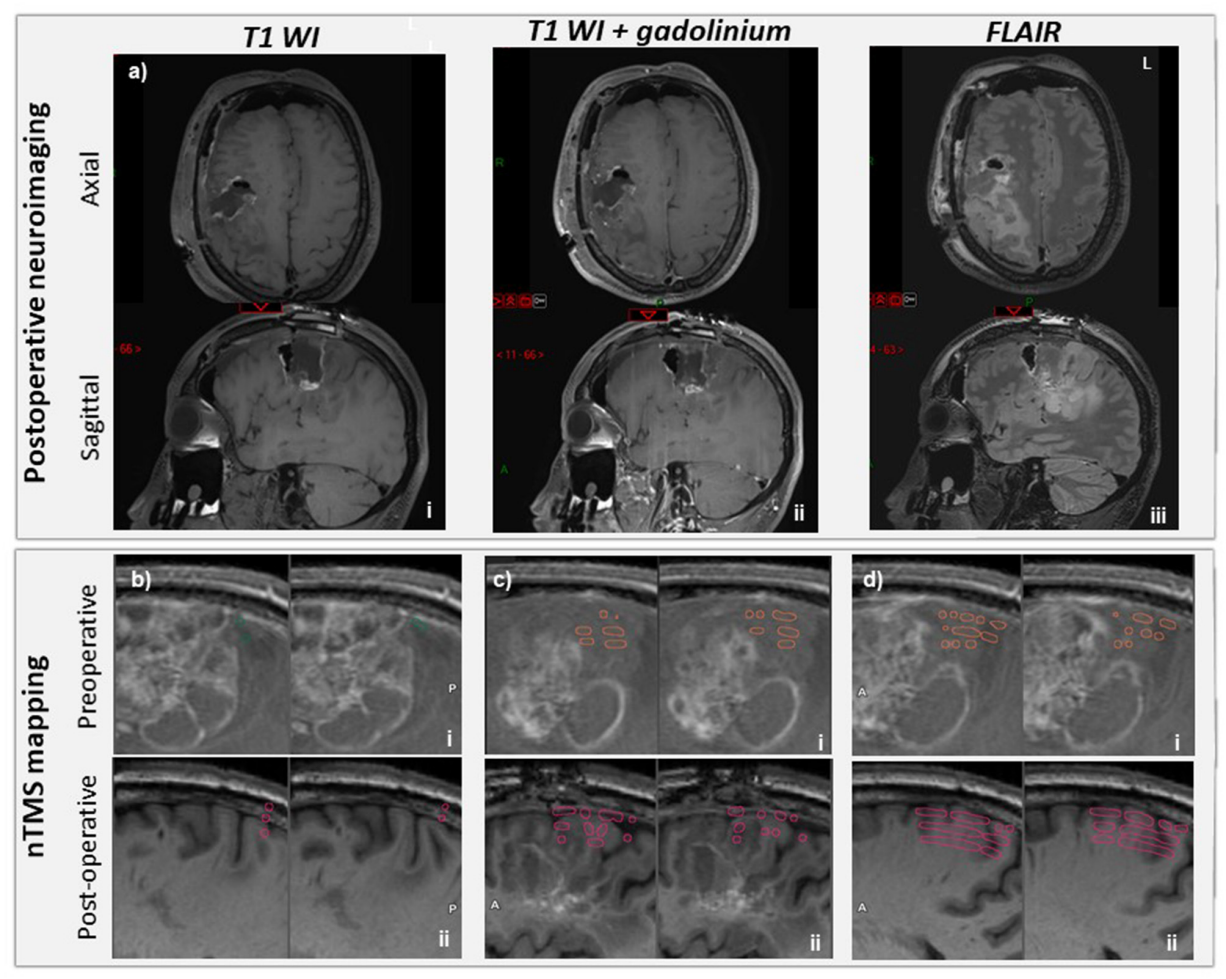
Post-operative neuroimaging status of the patient. **(a)** Day 1 postoperative MRI: axial (upper panel) and sagittal (lower panel) slices, T1WI (i), T1WI after gadolinium contrast injection (ii); FLAIR (iii). No residue with contrast enhancement was found. **(b)** Navigated transcranial magnetic stimulation (nTMS) mapping of left first dorsal interosseous (FDI) before (i, ROI in green) and 3 weeks after tumor resection (ii, ROI in pink). FDI nTMS map was shifted posteriorly 3 weeks after tumor resection. **(c)** nTMS map of left tibialis anterior (TA) before (i, orange) and 3 weeks after tumor resection (ii, pink) being larger with a posterior shift. **(d)** nTMS map of left deltoid before (i, orange) and 3 weeks after tumor resection (ii, pink) being larger with a posterior shift.

This procedure allowed a total resection of the tumor region with contrast enhancement to be performed, as well as a complete regression of IICP and dysarthria (Figure 4a). However, flair hypersignal with no contrast enhancement was persisting, especially at the posterior aspect of the resection cavity. It could not be removed during this surgery because of functional limits (Figure 4b). Histopathological examination of the tumor confirmed a grade III oligodendroglioma.

Hand paresis worsened slightly during the first post-operative week (2/5) and within 3 weeks it improved compared to the preoperative status (4/5). There was no additional deficit in the immediate postoperative time.

A post-operative nTMS mapping performed 3 weeks after the surgery confirmed the correct functional outcome, with a reorganization of the CM of the three muscles evaluated preoperatively (Figures 4b–d). Compared to preoperative assessment, TA-CM and deltoid-CM were moderately enlarged with a caudal-to-rostral spatial shift. On the other hand, the FDI-CM was reduced with a posterior-to-anterior shift, which justified performing an additional motor mapping for the abductor digiti minimi (ADM), abductor pollicis brevis (ABP) and the extensor carpi radialis (ECR) muscles. The CMs were performed at the same MSO for ADM and ABP than FDI (90%) on one hand, and for ECR than deltoid and TA (50%) on the other hand. The CM of these 3 additional muscles were significantly larger than the FDI-CM, and almost all the responses obtained were obtained for M1 regions distant from the residual tumorous lesion. These results allowed us to inform the patient about the need to focus on the FDI muscle for reinforcement exercises in physical therapy. These encouraging postoperative data led us to discuss the possibility of another resection surgery in the future, in order to prevent, or at least delay, further functional impairment of the patient's right upper limb.

## Discussion

This technical report underlines the value of preoperative functional and structural evaluation combining nTMS and various neuroimaging modalities (tractography and rs-fMRI) subsequently updated intraoperatively with BSC and elastic fusion between iCT and preoperative MRI. This multimodal approach may become the optimized technique of reference for patients with brain tumors in eloquent areas who do not qualify for awake brain surgery (Rath et al., 2014). By preserving some neuroanatomical ROIs, we make it possible to maintain or even improve key neurological functions, also opening perspectives for neurorehabilitation management and ultimately guarantee a certain level of quality of life for the patient.

### Pre-operative Mapping

Pre-operative cortical mapping with nTMS provides crucial functional information on cortical regions, specifying the anatomical relations between tumor location and eloquent brain regions. Although nTMS is not suitable to directly investigate deep subcortical structures due to its inherent physical limitations (Lefaucheur and Picht, 2016), subcortical mapping can be enabled by combining nTMS with function-guided tractography reconstructions of the corticospinal tract. Moreover, it is the most precise technique in the postoperative period to plan future resection surgery, giving valuable information about how cortical plasticity and rehabilitation might help to redesign the cortical motor map away from the resection cavity and prevent future relapse.

We did not achieve nTMS-guided speech mapping, although its negative (but not positive) predictive value is well-established in the literature (Picht et al., 2013). In fact, the patient was fatigued by the nTMS motor mapping session, which was performed first. Subsequently, the language assessment was planned but had to be interrupted because the picture naming task was too difficult to perform due to the reduction in the patient's attentional abilities and his fatigue. Nevertheless, it would be useful in the future to perform nTMS language mapping with appropriate patients and implement it in this pipeline.

On the other hand, we assessed the additional information provided by rs-fMRI, although we did not take this information into consideration for performing surgical resection, since its validity to guide surgical resection remains under evaluation (Brennan et al., 2016; Castellano et al., 2017; Ghinda et al., 2018). Indeed, caution should be exercised when rs-fMRI is used for surgical mapping, as the template atlases used for ROI-seed to compute rs-fMRI may have defects due to tumor-induced brain shift and brain plasticity with functional reorganization. We believe that rs-fMRI is still in the research stage and should not be used carelessly for surgical resection.

In fact, there are other non-invasive methods for mapping brain functions that could be implemented in our pipeline. For example, the use of electroencephalography (EEG) or magnetoencephalography (MEG) with source localization of sensory-evoked potentials or magnetic fields to map the sensorimotor cortex appears promising (Vitikainen et al., 2009). These non-invasive techniques could improve the relevance of preoperative mapping and be adjusted in the OR in combination with iCT-BSC.

### Discrepancies Between Pre-operative and Intraoperative Mapping With iCT: Improving Reliability During Operating Time With BSC

Spatial precision or resolution of nTMS mapping, tractography reconstructions after DWI, rs-fMRI are respectively on the order of 2–5, 1–3, and 2–4 mm (Glover, 2011; Schmidt et al., 2015; Holdsworth et al., 2019; Marquis et al., 2019) and their agreement with DES was found in the literature to be within 7.7, 8.7–10 mm (Berman et al., 2007; Takahashi et al., 2013; Cochereau et al., 2016). Furthermore, neuronavigation tracking errors in neurosurgery are ~1 mm (Gerard and Collins, 2015) and intraoperative brain shift can be as high as 20 mm and make neuronavigation even less precise and reliable (Gerard et al., 2017). In this case we found brain shifts of about 5 mm for the various anatomo-functional elements at risk (Figures 3c–e). On this basis, it was crucial to combine these preoperative anatomo-functional mapping techniques with intraoperative imaging using BSC since some key structures such as the corticospinal tract can be as thin as 2 mm in some of its portions, as observed in the present case. It was found that, without taking BSC into account, distances to speech tracts shorter than 8–11 mm can be associated with post-operative deficits (Sollmann et al., 2019). Thus, the spatial discrepancy between intraoperative DES and preoperative mapping, depending on the importance of the brain shift, is too high to guarantee a safety margin around the functionally important structures during the resection of brain tumors. This reinforces the need for BSC combined with intraoperative brain imaging to further reduce the risk of post-operative deficits. The use of BSC could significantly increase the spatial accuracy between pre-operative mapping and DES data. Future studies remain to be carried out to compare the respective values of the different preoperative mapping techniques (nTMS mapping, tractography reconstructions after DWI, rs-fMRI).

The use of iCT instead of intraoperative MRI as the intraoperative imaging modality was preferred in our pipeline since iCT is a shorter time-consuming technique and we expected to perform intraoperative brain imaging twice due to IICP. In fact, the surgical interruption when performing iCT-scan lasted 5 min, at both times, whereas intraoperative MRI scanning time ranges typically from 20 to 75 min (Chowdhury et al., 2018) and furthermore is not available in many centers due to its high cost and time-consuming use. There are several intraoperative imaging techniques and models for BSC (Valencia et al., 2012; Morin et al., 2017) but, from economical and practical points of view, the use of iCT may be easier to spread in the neurosurgical community since it can also be used for spine or stereotactic and deep brain stimulation surgical procedures (Scarone et al., 2018; Faust et al., 2019). However, iCT requires that the neurosurgeon be certified for this practice and for radiological protection. In this case, only a radiographer and not a radiologist, has to be available during the surgical procedure. The radiologist only needs to validate the quality and interpretation of iCT at the end of the surgery.

For centers that do not have Brainlab facilities, other methods could be used to compute BSC and implement preoperative mapping. For example, this can be done by re-performing surface mapping for neuronavigation along the exposed cortical veins (which rarely suffer from tumor shift) (Vitikainen et al., 2009) or by using ultrasound or laser range scanner for cortical surface registration to distort the preoperative MRI data according to the brain shift (Mäkelä et al., 2001). The respective performances of these various approaches remain to be compared.

In conclusion, the combination of preoperative mapping advanced techniques, asleep intraoperative motor nDES, and iCT with BSC and elastic fusion to preoperative brain MRI in a patient not eligible for awake craniotomy allowed for complete resection of tumor portions with contrast enhancement without permanent neurological deficits. Here, preoperative nTMS was only used to perform motor mapping, but our proposed pipeline could benefit in the future from preoperative nTMS mapping for speech, visual and cognitive functions.

## Conclusion: Patient Perspective

In our case report, the patient felt reassured to be operated following the M-iCT BSC procedure, which can be considered an “asleep awake-surgery” procedure. Although he did not benefit from AC, the patient could still feel involved in every step of the surgical treatment through our procedure. First, he could contribute to his surgery in optimal conditions: lying in a comfortable chair, skull closed, with the possibility to take some rest during the mapping session. Second, he could understand the exact surgical strategy, the anatomical, tractography and functional reconstructions being shown to him on a screen before surgery. He was fully aware of the risks and thus able to accept or refuse them. Third, on the basis of the postoperative nTMS mapping procedure, he better understood how to perform efficient neurorehabilitation to improve motor functions and also why a subsequent resection surgery might be eligible.

## Data Availability Statement

The original contributions presented in the study are included in the article/supplementary material, further inquiries can be directed to the corresponding authors.

## Ethics Statement

Written informed consent was obtained from the individual for the publication of any potentially identifiable images or data included in this article.

## Author Contributions

SS, BBar, and J-PL were responsible for the initial conception and draft of this manuscript. SS and BBar collected and analyzed data and prepared revised drafts and figures. BBar and SA carried out navigated TMS investigation. PB and BBap carried out neuroimaging investigation. SS, ST, KA, OL, KE, and SP were involved or responsible concerning the work-up of the patient, planning and conducting surgery, and providing clinical care. All authors contributed to the article and approved the submitted version.

## Conflict of Interest

The authors declare that the research was conducted in the absence of any commercial or financial relationships that could be construed as a potential conflict of interest.
